# The influence of electron utilization pathways on photosystem I photochemistry in *Synechocystis* sp. PCC 6803†

**DOI:** 10.1039/d2ra01295b

**Published:** 2022-05-16

**Authors:** Sharon L. Smolinski, Carolyn E. Lubner, Zhanjun Guo, Jacob H. Artz, Katherine A. Brown, David W. Mulder, Paul W. King

**Affiliations:** National Renewable Energy Laboratory 15013 Denver West Parkway Golden CO 80401 USA paul.king@nrel.gov

## Abstract

The capacity of cyanobacteria to adapt to highly dynamic photon flux and nutrient availability conditions results from controlled management and use of reducing power, and is a major contributing factor to the efficiency of photosynthesis in aquatic environments. The response to changing conditions includes modulating gene expression and protein–protein interactions that serve to adjust the use of electron flux and mechanisms that control photosynthetic electron transport (PET). In this regard, the photochemical activity of photosystem I (PSI) reaction centers can support balancing of cyclic (CEF) and linear electron flow (LEF), and the coupling of redox carriers for use by electron utilization pathways. Therefore, changes in the utilization of reducing power might be expected to result in compensating changes at PSI as a means to support balance of electron flux. To understand this functional relationship, we investigated the properties of PSI and its photochemical activity in cells that lack flavodiiron 1 catalyzed oxygen reduction activity (ORR1). In the absence of ORR1, the oxygen evolution and consumption rates declined together with a shift in the oligomeric form of PSI towards monomers. The effect of these changes on PSI energy and electron transfer properties was examined in isolated trimer and monomer fractions of PSI reaction centers. Collectively, the results demonstrate that PSI photochemistry is modulated through coordination with the depletion of electron demand in the absence of ORR1.

## Abstract 2

Alteration of electron utilization pathways affects PSI reaction center structure and photochemical properties.

## Graphical abstract

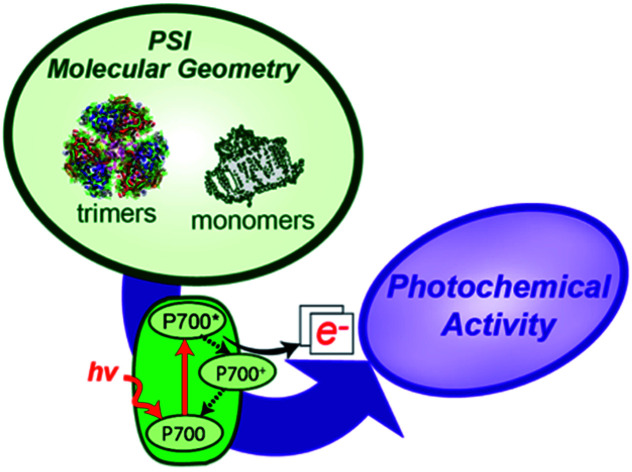

## 1 Introduction

Oxygenic photosynthesis is the process that converts sunlight into chemical energy important for sustaining life. The light reactions are carried out by the photosystem II (PSII) and photosystem I (PSI) reaction centers which utilize photons to generate the reducing equivalents necessary for chemical bond formation.^1,2^ Reaction centers thus represent an important control point where the flux of photons and electrons is coordinated and managed.^3^ In particular, PSI integrates with a network of redox partners to distribute reducing equivalents among various pathways depending on the balance of energy input and metabolic requirements. When this balance is perturbed, a variety of compensating mechanisms are employed, for example state transitions which adjust the distribution of photon energy between the PSII and PSI reaction centers. This dynamic response allows cells to quickly react to rapidly changing conditions. However, effects of energy mismatch on the structural, photochemical, and electron transfer processes within reaction centers are less well understood.

Photooxidation of cyanobacterial PSI has been reported to be affected when electron consuming pathways peripheral to photosynthesis are disrupted, presumably due to acceptor side limitations in electron transfer at PSI.^4–8^ These pathways act as sinks for electrons under certain environmental and physiological conditions, and disruptions to these typically result in electron congestion at the PSI acceptor side.^9^ One such pathway comprises flavodiiron (Flv) enzymes which catalyze the oxygen reduction reaction (ORR) that contributes to the relief of excess electron flux *via* the generation of water.^6,10^ In whole cells, the PSI redox state is significantly more reduced in flv1 and flv3 deletion strains that have a severe loss in ORR activity, compared to wild-type strains.^6^ Changes in PSI redox state are known to be linked to changes in function and interactions with photosynthetic components, suggesting that as peripheral electron flux is modulated by growth conditions, there is a corresponding compensation in function of PSI. Understanding PSI function in mediating photosynthetic transport in strains where the electron flux and utilization pathways are changed, for example by addition of CO_2_ utilizing enzymes,^11–14^ or removal of reduction reaction enzymes,^15,16^ is a means to gain deeper insight into how PSI activity is controlled. A lack of understanding of the broader response of PSI function within these contexts, as well as the impact on the activities and functions of other photosynthetic electron transport components, limits the potential of engineering cyanobacteria for biotechnological applications. Additionally, it obscures a fundamental understanding of how PSI has evolved to control electron flow.

Here, we have employed the ORR1 strain of *Synechocystis* sp. PCC 6803 (*S.* 6803) to probe the physical and electronic modifications that occur at PSI when an electron consuming pathway is lacking, in order to gain insights into the complex interplay of downstream reactions and primary photochemical events. Specifically, we investigated the effects of diminished electron flow through peripheral pathways in the ORR1 strain on the structural, photochemical and electron transfer properties of the PSI reaction center. The results demonstrate that the loss of ORR1 leads to profound changes in a broad spectrum of PSI properties that collectively down regulate PSI photochemistry.

## 2 Experimental

### 2.1. Strains and culture conditions

*Synechocystis* sp. PCC 6803 (*S.* 6803) WT and a strain deficient in ORR (oxygen reduction reaction), ORR1, were used for experimentation. The ORR1 strain was generated by knocking out the flv1 gene, by transforming fusion PCR fragments into WT. Primers designed with a 15-bp overlap (5-bp) were used to amplify upstream and downstream regions of flv1 gene from WT genomic DNA. Spectinomycin and kanamycin resistance cassettes, amplified from pJU158 and pUC4K respectively, were used to complete the construction of the PCR fragments to disrupt expression of flv1. The resulting 1.711 kb fusion PCR product was used for direct transformation of the WT strain, and segregation was monitored using PCR (see Table S1 in ESI†).

WT and ORR1 strains were maintained on BG-11 (ref. 17) supplemented with 20 mM NaHCO_3_, 20 mM TES (pH 6.8), and appropriate antibiotics (25 μg l^−1^ spectinomycin for ORR1). Maintenance cultures were grown in a Percival chamber in 250 ml flasks at 30 C, 50 μmol photons per m^2^ per s white LED light, in air supplemented with 5% CO_2_ and with shaking (120 rpm). For experimental conditions, two light regimes were used: a standard condition of 35 μmol photons per m^2^ per s continuous growth light (GL), and increased photon flux using fluctuating light (FL) (cycles of 35 μmol photons per m^2^ per s for 5 min and 500 μmol photons per m^2^ per s for 30 s). Strains were first grown under GL for 36 h, then were either continued under GL or switched to growth under FL for a period of 96 h. Cultures were grown in custom NREL bioreactor bottles, 40 cm tall by 13 cm wide cylindrical glass with a round bottom and narrow neck fitted with a rubber stopper and tubing for gas delivery and sampling. Cultures were grown at room temperature, in air supplemented with 3% CO_2_, agitated using stirring (0.5 inch stir bar) and bubbling, and were illuminated from the side using white LED panels.

### 2.2. Isolation of thylakoid membranes

Thylakoid membranes (TMs) were isolated from cells similar to literature.^18^ Cells were harvested by centrifugation at 5000 × *g* for 10 min at 4 °C, and the pellet was resuspended in 20 mM HEPES–NaOH buffer, pH 7.5, with 10 mM CaCl_2_, 10 mM MgCl_2_, 10 mM NaCl, and 15% glycerol. Cells were lysed using three passes through a French Press cell, while samples were kept on ice. Cell lysate was then centrifuged at 5000 × *g* for 10 min at 4 °C, and the supernatant was collected and then ultracentrifuged at 208 000 × *g* for 1 h at 4 °C (Type 70 Ti rotor, Beckman Coulter). The pellet containing TMs was resuspended in the same buffer to approximately 1 mg chl per ml. TMs were solubilized in buffer containing *n*-dodecyl-β-d-maltoside (DDM) (DDM25, GoldBio), at a final concentration of 1%, and chl concentration was adjusted to 0.4–0.5 mg ml^−1^. Samples were solubilized by stirring gently for 2 h at 4 °C in darkness. Solubilized TMs were centrifuged at 12 000 × *g* for 20 min at 4 °C to pellet non-solubilized material, and the supernatant was collected. Isolated TMs were frozen in the same buffer in liquid N_2_ and stored at −80 °C.

### 2.3. Isolation of PSI trimers and monomers

To isolate PSI trimers and monomers using anion-exchange chromatography, solubilized TMs were loaded onto a packed column of DEAE (17-0709-01, GE Healthcare), with a column volume (CV) of 150 ml, on a chromatography system (AKTA Purifier 10), run with Unicorn software (GE Healthcare). A flow rate of 4–5 ml min^−1^ was used for all steps, except the sample was loaded onto the column at 3 ml min^−1^. The column was equilibrated with 3 CVs of equilibration buffer (20 mM HEPES–NaOH buffer, pH 7.5, with 10 mM CaCl_2_, 10 mM MgCl_2_, 10 mM NaCl, and 0.04% DDM). After solubilized TMs were loaded onto the column using equivalent amounts of chl (2 mg), the column was washed with 2 CVs of equilibration buffer. Fractions containing PSI monomers and PSII, and PSI trimers were eluted with 20 mM HEPES buffer, pH 7.5, with 10 mM CaCl_2_, 10 mM MgCl_2_, 10 mM NaCl, 0.04% DDM and 500 mM MgSO_4_ using a linear gradient of 0 mM to 300 mM MgSO_4_, followed by an increase to 500 mM MgSO_4_ (Fig. S1†), similar to ref. 19. Eluted samples were dialyzed against equilibration buffer for 16 h at 4 °C (132542, Spectrum Labs). Samples were concentrated using centrifugal filtration units (901024, 503096, EMD Millipore). Samples were frozen in equilibration buffer containing 10% glycerol in liquid N_2_ and stored at −80 °C.

To isolate PSI trimers and monomers using sucrose gradients, solubilized TMs were centrifuged at 12 000 × *g* for 20 min to remove non-solubilized material. Then the supernatant was loaded onto a 5–30% (w/v) linear sucrose gradient with 20 mM HEPES–NaOH, pH 7.5, with 10 mM CaCl_2_, 10 mM MgCl_2_, 10 mM NaCl, and 0.01% DDM. The gradient was centrifuged at 110 000 × *g*, overnight. Each colored fraction was carefully collected from the top of the sucrose gradient. The PSI monomer/PSII and PSI trimer fractions were further concentrated by 10 kDa cutoff filter (Amicon Ultra-15, 10 kDa cutoff, Sigma) at 4 °C and then flash frozen in liquid nitrogen, stored at −80 °C. PSI fractions isolated from sucrose gradients were analyzed using BN-PAGE (Fig. S2†) and used for fluorescence emission analysis of isolated PSI.

### 2.4. BN-PAGE

Fractions isolated using anion-exchange chromatography were analyzed using BN-PAGE. Samples were in buffer containing 20 mM HEPES–NaOH, pH 7.5, with 10 mM CaCl_2_, 10 mM MgCl_2_, 10 mM NaCl, and 0.04% DDM. Samples were measured for chl concentration, and equivalent amounts of chl were suspended with sample buffer (BN2003, Thermo Fisher Scientific) and 0.1 volumes of 5% G250 (BN2004, Thermo Fisher Scientific). Since the fractions were prepared in buffer containing DDM, additional DDM was not introduced to the sample buffer. Samples were loaded onto 4–16% Bis/Tris gels (BN1002BOX, Thermo Fisher Scientific) alongside standards (57030, Invitrogen), and run in a Mini Gel Tank (A25977, Thermo Fisher Scientific) at 150 V for 2 h in Bis/Tris running buffer (BN2001, Thermo Fisher Scientific). Resulting bands were compared to literature.^20,21^

### 2.5. Determination of PSI trimer to monomer ratio

The relative amounts of PSI trimers and PSI monomers isolated using anion-exchange chromatography were determined using the peak areas of absorbance at 700 nm, using Unicorn software (v. 5.1) (Tables S2 and S3†). The ratio of PSI trimers to PSI monomers was calculated as (PSI trimer *A*_700_ peak area) ÷ (PSI monomer *A*_700_ peak area). PSI trimer and PSI monomer as a percentage of total PSI was based on the areas of the respective fractions, as (peak area of PSI monomer) ÷ (peak area of PSI monomer + peak area of PSI trimer) × 100.

### 2.6. Fluorescence emission analysis

Fluorescence emission spectra were measured at room temperature and at 77 K with a Fluorolog-3 spectrometer (HORIBA Scientific). Fractions containing PSI trimers and monomers that were isolated using anion-exchange chromatography were normalized to 3.0 mg chl per ml and were measured at 77 K to quantify P_700_. Fractions containing PSI monomers and trimers that were isolated using sucrose gradients were normalized to 16 mg chl per ml and were measured at room temperature and 77 K to determine spectral properties. An excitation wavelength of 450 nm was used, and emission spectra were collected between 600 nm and 850 nm. The excitation slit is 5 nm and emission slit is 1 nm. The samples (200 μl) were held inside a 4 mm EPR tube. The ratio of emission maxima at 685 to 695 nm and 718 to 722 nm were used to calculate the PSII to PSI ratio.

### 2.7. P_700_ spectroscopic analysis

P_700_ spectroscopic analysis on whole cells was performed using a JTS-10 spectrophotometer (Spectrologix) equipped with a LED light emitting 630 nm actinic light, with 705 nm interference filters. Each sample was measured using 2000 μmol photons per m^2^ per s actinic light, over 8 cycles separated by 30 s and averaged. Cells were collected from cultures and normalized to 5 μg chl per ml, and measurements were conducted with and without inhibitors. The inhibitor 3-(3,4-dichlorophenyl)-1,1-dimethylurea (DCMU) was added to cell samples to eliminate the contribution of electrons from PSII^22,23^ at a final concentration of 10 μM. Methyl viologen (MV) was added to cell samples to alleviate PSI acceptor side limitations by serving as a terminal electron acceptor,^24,25^ at a final concentration of 500 μM, with incubation for 3 min prior to measurement. The value used for steady state oxidation is the change in absorbance at 705 nm during illumination just prior to the transition to darkness. The value for the half-life of dark re-reduction was calculated from fits using either a linear (for MV data and no inhibitor WT data) or a single exponential equation (for no inhibitor ORR strain data and DCMU data). The linear equation: *y* = *mx* + *b*. The single exponential equation: *y*(*t*) = *A*_0_ × exp(*kt*) + *c*. The equation for half-life: *t*_1/2_ = ln(2)/*k*. In the absence of inhibitors, the ORR1 strain exhibited an initial linear region upon darkness which was not included and the rate was calculated based on the subsequent exponential portion of the curve. Curves were fit to minimize the sum of square deviations.

P_700_ spectroscopic analysis on isolated fractions containing PSI monomers or trimers were normalized to equivalent amounts of P_700_ (0.84 μmol) and measured using 720 nm actinic light. Samples (2 ml) were placed in a quartz cuvette containing P_700_, 10 mM sodium ascorbate, and 10 mM 2,6-dichlorophenolindophenol (DCPIP) in 20 mM HEPES–NaOH, pH 7.5, with 10 mM CaCl_2_, 10 mM MgCl_2_, 10 mM NaCl, and 0.04% DDM. Samples were allowed to equilibrate to darkness for 1 min. Baseline P_700_ absorbance was measured using pulses over 10 s before illumination. Samples were then illuminated for 5 s with 200 or 250 to 5000 μmol photons per m^2^ per s actinic light, followed by 8 s of darkness. The value for steady state oxidation is the change in absorbance at 705 nm during illumination just prior to the transition to darkness. The value for the half-life of oxidation and dark re-reduction was calculated from fits using a single exponential equation, as described above, using data from 10.00 to 14.31 s and 15.00 to 18.98 s, respectively.

### 2.8. Flavodoxin photoreduction assays

In order to determine the capacity of PSI monomers and trimers to transfer electrons out of PSI, equivalent amounts of P_700_ (44 nmol) were added to a quartz cuvette containing 10 mM sodium ascorbate, 30 mM phenazine methosulfate, and 100 mM flavodoxin (Fld), in 20 mM HEPES–NaOH, pH 7.5, with 10 mM CaCl_2_, 10 mM MgCl_2_, 10 mM NaCl, and 0.04% DDM, at a final volume of 350 ml, similar to ref. 26. Solutions were added to the cuvette in near darkness, and these were incubated in darkness for 10 min prior to measurement. Absorbance at 580 nm was measured for 2 min in darkness, then for 2 min under illumination with a 660 nm LED light at an intensity of approximately 530 μmol photons per m^2^ per s. Data were collected for two to five technical replicates. Rates of Fld reduction were calculated using a least squares regression to determine the fastest initial rate over a 1 s range and baseline corrected. Flavodoxin (isiB) from *Synechococcus* sp. PCC 7002 was recombinantly expressed and purified according to Zhao *et al.*^27^ Fld concentration employed in these assays was chosen based on a linear initial response after illumination.

## 3 Results and discussion

### 3.1. Increased monomerization of PSI accompanies decreased electron utilization

**Fig. 1:**
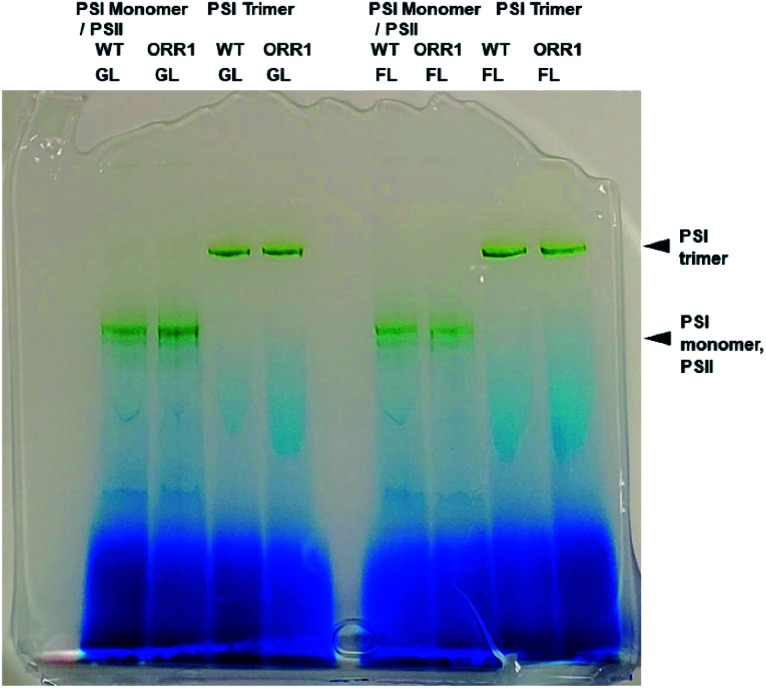
BN-PAGE analysis of PSI fractions purified from thylakoid membranes using anion-exchange chromatography from WT and ORR1 cells grown in standard conditions (GL) or under increased light intensity (FL).

To confirm the effect of the loss of ORR1 on photosynthetic electron transport (PET), the O_2_ uptake and O_2_ evolution activities of wild-type and ORR1 strains were measured. In the absence of ORR1 there was a complete loss of ^18^O_2_ uptake activity and a 44% decrease in O_2_ evolution (Table S4†) compared to the WT strain. The decline in O_2_ evolution rate in ORR1 was not a result of a difference in PSII abundance since PSI-to-PSII ratios were similar between the two strains (Table S5†). To examine the consequences of O_2_ transport changes on PSI photochemistry, the total fraction of PSI reaction centers including trimers and monomers were isolated from *S.* 6803 WT and ORR1 cells. When the PSI-containing thylakoid membrane fraction was separated on sucrose gradients, the ratio of PSI monomers to trimers was observed to be slightly higher in ORR1, which was confirmed by further isolation of the monomeric and trimeric PSI fractions using anion exchange chromatography (Fig. S1†) and separation by BN-PAGE (Fig. 1). The chromatography data demonstrated that the relative *A*_700_ peak area values of the PSI monomer fraction were higher than the trimer fraction (Tables 1, S2 and S3†). An analysis of the PsaL subunit levels in the PSI enriched fractions was performed to determine whether monomerization was associated with any changes in the PsaL abundance, since this subunit is necessary for trimer formation and stability.^28^ PSI fractions from WT and ORR1 cells were separated by SDS-PAGE and probed by immunoblots to PsaL antibodies (Fig. S3†). The results indicated there were no differences in PsaL levels among monomer or trimer PSI fractions from either WT or ORR1 strains, indicating that the increase in PSI monomers in ORR1 did not result from a decrease of PsaL.

**Table 1:** PSI oligomer compositions in WT and ORR1 grown under standard conditions

Strain	Trimer : monomer^a^	Monomer : trimer^b^	Monomer^c^ (% of total PSI)
WT	6.3 ± 1.9	0.17 ± 0.05	14 ± 0.03
ORR1	4.6 ± 1.2	0.23 ± 0.07	18 ± 0.04

a Values based on 2 mg chl of thylakoid membrane samples loaded onto DEAE column and separated by anion-exchange chromatography, see Table S2. PSI trimer ÷ PSI monomer. Values based on the average of three biological replicates ± standard deviation.

b Values obtained the same as for “a”, except PSI monomer ÷ PSI trimer.

c PSI monomer as a percentage of the total PSI based on areas of fractions, calculated as (peak area of PSI monomer) ÷ (peak area of PSI monomer + peak area of PSI trimer) × 100.

### 3.2. P_700_ kinetics and photooxidation capacity of PSI

**Fig. 2:**
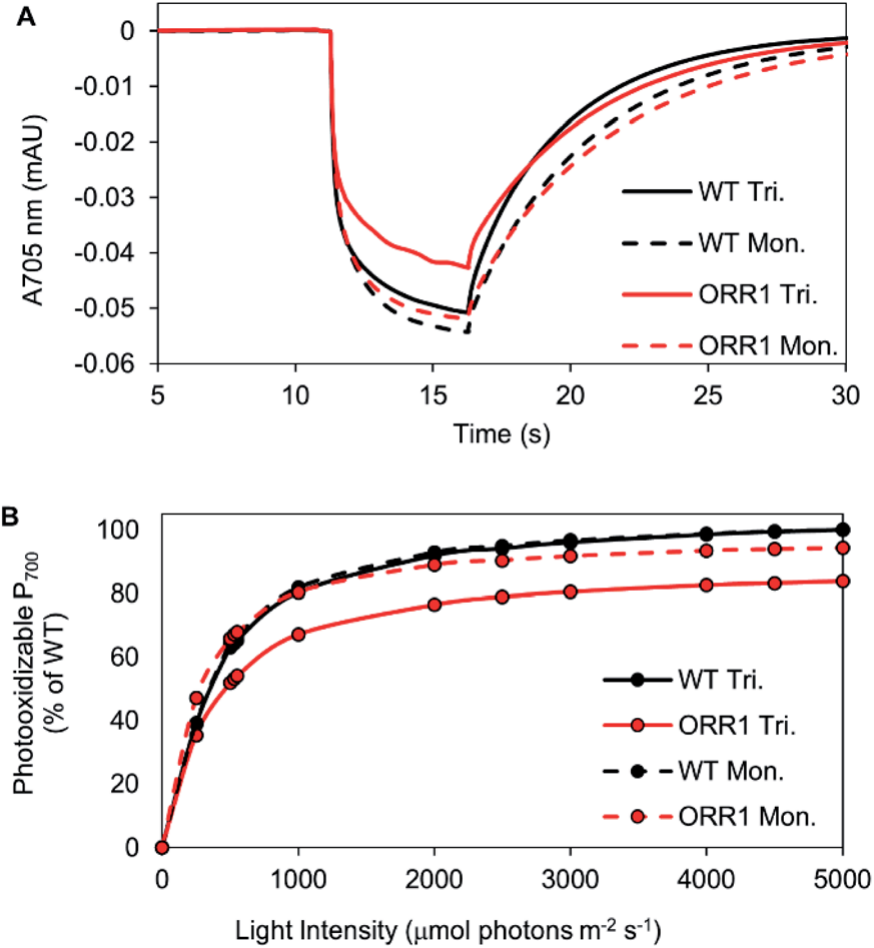
Photochemical properties of PSI reaction centers isolated from WT and ORR1 strains. (A) P_700_ kinetic traces of isolated PSI monomers and trimers from WT and ORR1, measured at 2000 μmol photons per m^2^ per s. (B) Light saturation curves of PSI monomers and trimers from WT and ORR1, normalized to equivalent P_700_ concentrations. Maximal P_700_ photooxidation amplitude was plotted against actinic light intensity (200 to 5000 μmol photons per m^2^ per s). Values were normalized to maximum WT photooxidation (trimers were normalized to WT trimers, monomers were normalized to WT monomers). WT: black, ORR1: red, PSI trimers: solid line, PSI monomers: dashed line.

Since the ORR1 strain is diminished in both O_2_ evolution and consumption rates compared to WT, this indicates that the flux of electrons through the photosynthetic chain is altered, possibly impacting the photochemical properties of PSI. To investigate the fidelity of the PSI reaction centers, P_700_ photooxidation activity was measured. WT monomers and trimers as well as ORR1 monomers achieved similar maximum oxidation amplitudes, however ORR1 trimers displayed a slight loss in P_700_ oxidation ability (measured at 2000 μmol photons per m^2^ per s of 720 nm actinic light) (Fig. 2A). Light saturation curves were produced to determine the effect of light intensity on P_700_ photooxidation activity (200 to 5000 μmol photons per m^2^ per s actinic light). Distinctive changes were observed for PSI trimers from ORR1, which reached saturation at lower light intensities (Fig. 2B, Table S6†). The photochemical profiles of isolated PSI reaction centers suggest changes to the photon capture and conversion processes of PSI trimers from ORR1.

Additionally, this decrease in the level of P_700_ photooxidation was also evident in whole cells of ORR1 (Fig. S4, Table S7†). Furthermore, the decreased dark re-reduction rates in ORR1 whole cells reflects a PSI acceptor side limitation due to the loss of ORR1 activity (Fig. S4A, Table S8†), consistent with previous studies.^6,7^ Upon the addition of 3-(3,4-dichlorophenyl)-1,1-dimethylurea (DCMU) to eliminate the contribution of electrons from PSII^22,23^ (Fig. S4B, Table S8†), a 2.5-fold faster rate of dark re-reduction was observed in the ORR1 strain indicating increased CEF. While these properties have been observed in whole cells previously,^7^ the reasons underlying this behavior have not been unambiguously assigned before now.

### 3.3. Spectral properties of PSI

**Fig. 3:**
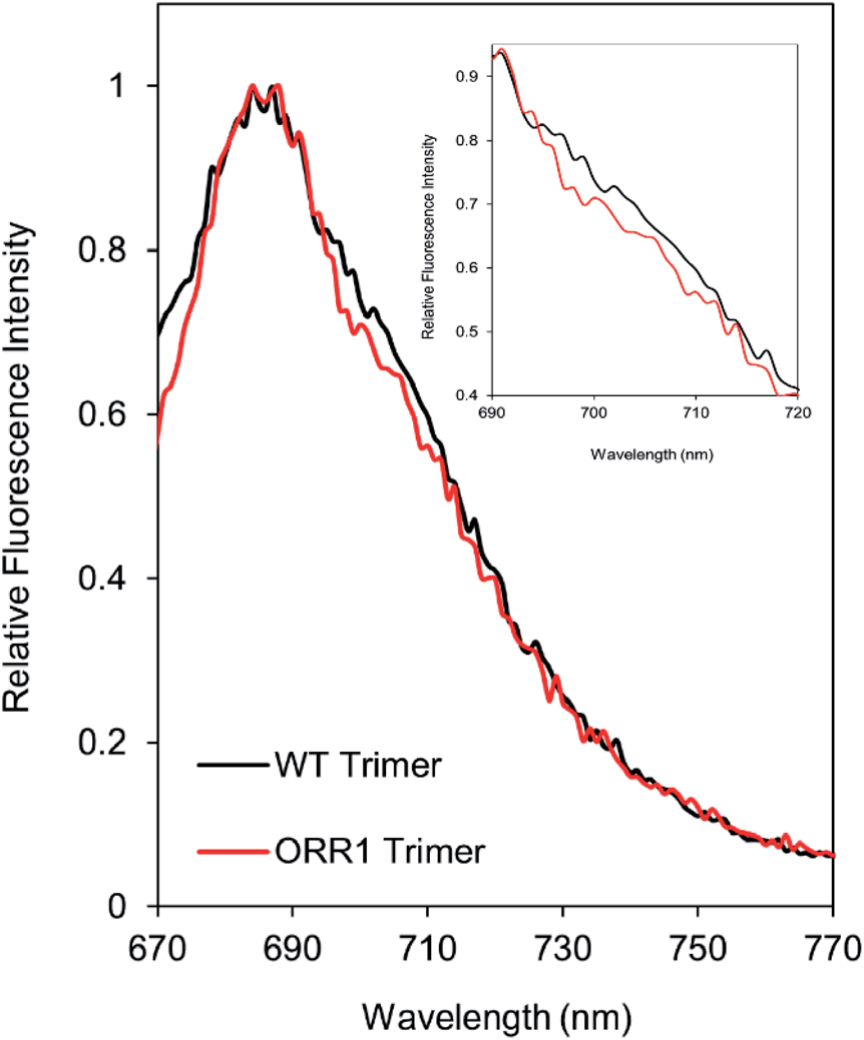
Fluorescence emission spectra of PSI trimers. Fluorescence emission spectra collected at room temperature using 450 nm excitation on PSI trimers isolated from WT and ORR1 cells grown in standard conditions. Inset shows a magnification of the region from 690 nm to 720 nm, showing the decreased shoulder for ORR1 PSI trimers indicative of decreased contributions from red chls. Data were normalized to the maximum peak (684 nm). Peak intensity was normalized to WT trimer for each condition. WT: black, ORR1: red.

Due to the decreased P_700_ photooxidation in PSI trimers in the ORR1 strain, the spectral properties of PSI monomers and trimers were assessed. In *S.* 6803, the long-wavelength (red) chls contribute to the room temperature fluorescence emission spectra of purified PSI in the shoulder region around 710 nm^29,30^ and may contribute to small peak shifts in the 77 K spectra at 720 nm.^31^ Room temperature fluorescence emission spectra of PSI trimers isolated from ORR1 displayed losses in the shoulder region between approximately 700 and 720 nm (Fig. 3). These findings suggest a relationship between spectral changes of the PSI antenna and photochemistry of the reaction center arising from the loss of ORR1. Significant losses in this shoulder region were also found in the spectra of PSI monomers from both WT and ORR1 (Fig. S5A†), which was anticipated as it is well known that monomerization results in changes to the red chl environment.^31^

### 3.4. Electron transfer out of PSI

Given alterations in the spectral properties and generation of photoexcited electrons at PSI, we examined the capacity for electron transfer beyond PSI. Since oxidized flavodoxin (Fld) accepts electrons directly from PSI,^32^ we measured the rates of photo-driven Fld reduction. Rates of Fld reduction by PSI were measured spectrophotometrically using equivalent amounts of P_700_. Both the PSI monomers and trimers that were isolated from ORR1 showed a ∼10% lower rate of Fld photoreduction compared to their WT counterparts (Table 2). Overall, the results demonstrate that compensation for the loss of ORR1 affects PSI through decreases in P_700_ photooxidation, changes in spectral properties of the antenna, and a relatively minor effect on electron transfer rates.

**Table 2:** Rates of Fld photoreduction with PSI monomers or trimers isolated from WT and ORR1 cells grown under standard conditions

Strain	PSI	Rate of Fld reduction^a^	Rate of Fld reduction (% of WT)
WT	Trimer	29 ± 1.9	100
ORR1	Trimer	27 ± 1.1	93
WT	Monomer	26 ± 4.5	100
ORR1	Monomer	23 ± 0.02	88
WT	Monomer	0.05 (*no Fld added)	0.8
NA	NA	0.02 (*no PSI added)	0.3

a Rate of Fld reduction as (μmol Fld per μmol P_700_ per h). Values are the average of two to three replicates ± standard deviation, except controls.

### 3.5. PSI properties under increased photon flux

Cyanobacteria frequently experience dramatic changes in incident light quality and quantity within an environmental setting, which can contribute to alterations in electron generation and utilization. Because expression of ORR1 is upregulated under conditions of high and/or fluctuating light (FL),^33^ we investigated whether additional effects on PSI photochemical properties were enacted under this light regime. When irradiant light was increased during growth (5 min of 35 μmol photons per m^2^ per s, 30 s of 500 μmol photons per m^2^ per s) lower rates of O_2_ evolution were observed in ORR1 but not in the WT strain (Table S9†). In contrast to the standard light conditions employed above, a decrease in growth rate of ORR1 was observed (Fig. S6†), suggesting a greater impact to the coordination of electron generation and utilization. Increases in PSI monomer were again observed for ORR1, albeit to a greater extent (∼5-fold more than WT, Table 3). The increased excitation pressure had no apparent effect on the presence of the PsaL subunit, which was found in PSI monomers and trimers from both strains (Fig. S3†).

**Table 3:** PSI oligomer compositions in WT and ORR1 grown under increased photon flux

Strain	Trimer : monomer^a^	Monomer : trimer^b^	Monomer^c^ (% of total PSI)
WT	9.2 ± 1.1	0.11 ± 0.01	10 ± 1
ORR1	2.0 ± 0.5	0.53 ± 0.15	34 ± 6

a Values based on 2 mg chl of thylakoid membrane samples loaded onto DEAE column and separated by anion-exchange chromatography, see Table S2. PSI trimer ÷ PSI monomer. Values based on the average of three biological replicates ± standard deviation.

b Values obtained the same as for “a”, except PSI monomer ÷ PSI trimer.

c PSI monomer as a percentage of the total PSI based on areas of fractions, calculated as (peak area of PSI monomer) ÷ (peak area of PSI monomer + peak area of PSI trimer) × 100.

**Fig. 4:**
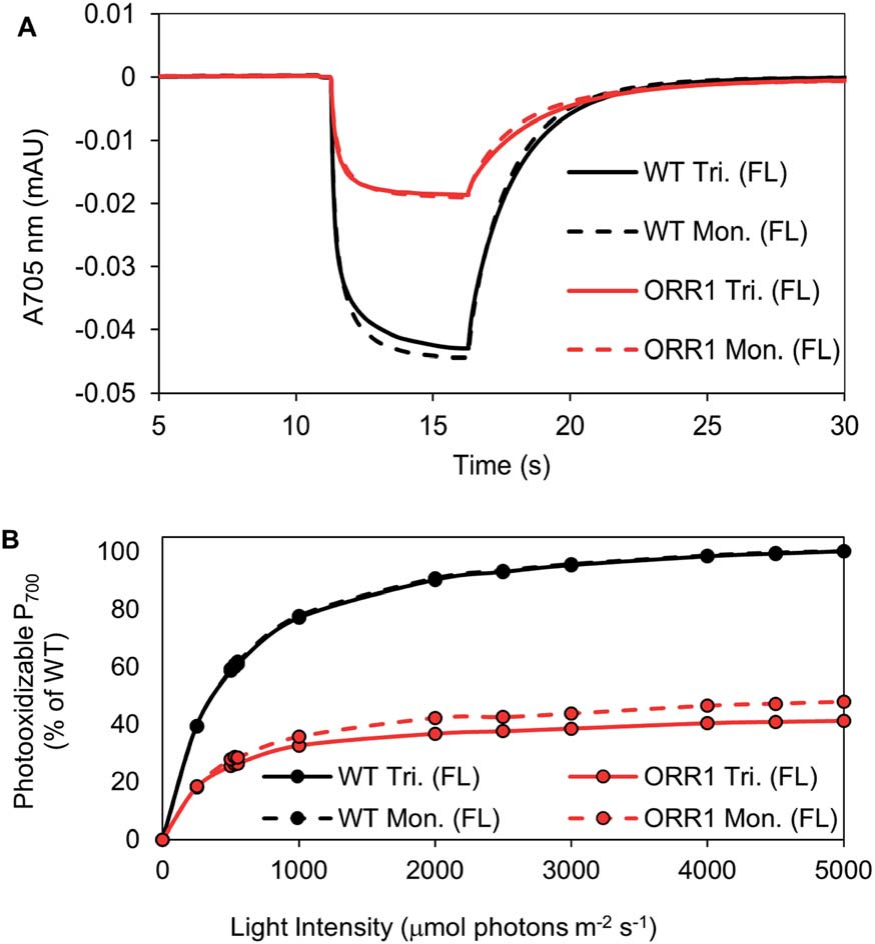
P_700_ kinetic traces and light saturation curves of P_700_ photooxidation of isolated PSI monomers and trimers, from cells grown under increased photon flux (FL). PSI was isolated from WT and ORR1 cells using anion-exchange chromatography. (A) The kinetic trace of P_700_ at 2000 μmol photons per m^2^ per s. Measurements used equivalent P_700_. (B) The maximum value of P_700_ photooxidation was measured at different actinic light intensity (200 to 2000 μmol photons per m^2^ per s). Values were normalized to maximum WT photooxidation (trimers were normalized to WT trimers, monomers were normalized to WT monomers). WT: black, ORR1: red, PSI trimers: solid line, PSI monomers: dashed line.

P_700_ photooxidation and light saturation profiles of the isolated PSI reaction centers were measured. ORR1 monomers and trimers were found to saturate at lower light intensities (2.5-fold lower *vs.* WT) (Fig. 4, Table S6†). The decreased amplitude of P_700_ oxidation was also evident in whole cells (at 2000 μmol photons per m^2^ per s actinic light) (Fig. S7, Table S7†), and the addition of the artificial electron acceptor methyl viologen (MV) led to only a partial recovery of P_700_ photooxidation (Fig. S8, Table S7†). These results demonstrate that under higher excitation pressure and in the absence of ORR1, there is less capacity of PSI to generate photoexcited electrons than under standard light conditions (Tables S7 and S8†). This property may lead to a greater attenuation of electron transfer from PSI to external electron carriers. Indeed, the light-driven Fld reduction rates by ORR1 PSI were on average 71% of the WT rates (Table 4). Increased excitation pressure additionally led to faster rates of P_700_ re-reduction in whole cells in the presence of DCMU (Fig. S9, Table S7†) consistent with a further increased level of CEF.

**Table 4:** Rates of Fld photoreduction with PSI monomers or trimers isolated from WT and ORR1 cells grown under increased photon flux

Strain	PSI^a^	Rate of Fld reduction^a^	Rate of Fld reduction (% of WT)
WT	Trimer	27 ± 1.0	100
ORR1	Trimer	18 ± 1.5	67
WT	Monomer	24 ± 5.2	100
ORR1	Monomer	18 ± 0.2	75

a Rate of Fld reduction as (μmol Fld per μmol P_700_ per h). Values are the average of three to five replicates ± standard deviation.

**Fig. 5:**
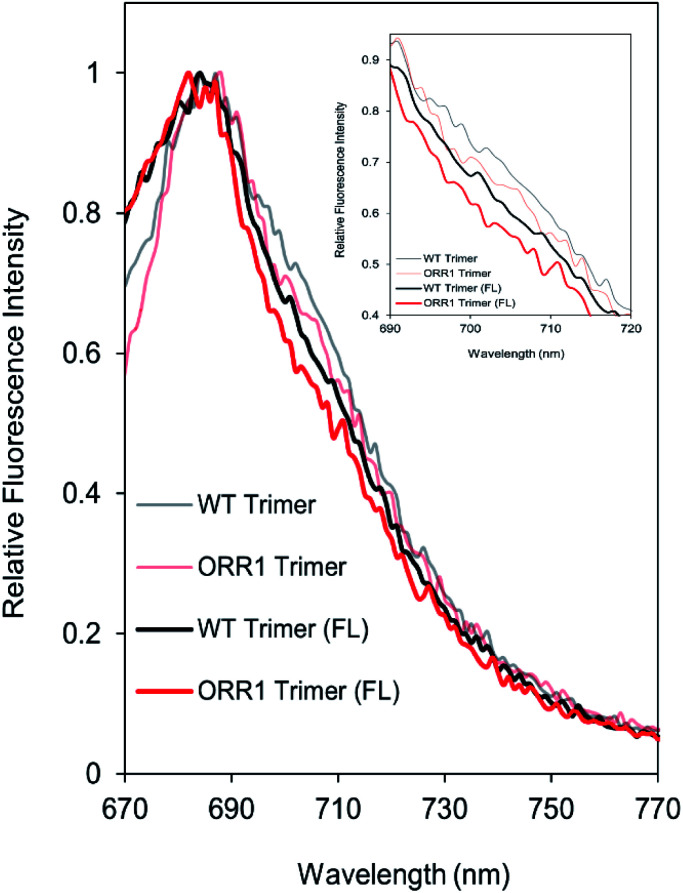
Fluorescence emission spectra of PSI trimers. Fluorescence emission spectra collected at room temperature using 450 nm excitation on PSI trimers isolated from WT and ORR1 cells grown in standard conditions or increased photon flux (FL). Inset shows a magnification of the shoulder region from 690 nm to 720 nm indicative of decreased contributions from red chls. Data were normalized to the maximum peak (684 nm for WT GL, ORR1 GL, WT FL; and 682 nm for ORR1 FL). WT: black, ORR1: red, standard growth conditions: transparent line, FL: solid line.

The spectral properties of PSI monomers and trimers isolated from ORR1 showed greater alterations in the contributions from red chls when cells were grown under increased photon flux (Fig. 5). Under this light regime, isolated PSI trimers from both WT and ORR1 exhibited a decrease in red chl contributions in the RT and 77 K fluorescence spectra. The room temperature spectra show a decreased shoulder region for fractions containing PSI monomers in both WT and ORR1 (Fig. S5†), similar to literature which has reported decreased red chls in PSI monomers.^31^ Interestingly, the ORR1 trimers displayed more significant peak losses in the shoulder around 700–720 nm at RT (Fig. 5) and the PSI peak around 722–724 nm, which was also blue-shifted from 719 nm to 717 nm in the 77 K fluorescence spectra (Fig. S10†). Overall, the results demonstrate a decreased contribution from red chls in PSI trimers isolated from ORR1, and for PSI monomers in both WT and ORR1. Thus, in addition to changes in the photochemical properties of PSI that include increased CEF, a shift to monomerization, and lower P_700_ photooxidation there are changes to the photoexcitation properties that combine to dramatically alter PSI function in mediating electron flow to match changes in electron utilization.

## 4 Discussion

PSI serves as an integral component of the PET chain, yet the specific consequences for PSI structure and function when electron utilization is altered have not been fully elucidated. To help address this gap, we employed a strain that is hindered in the ability to perform ORR in order to provoke a condition where electron utilization is mismatched with electron generation. This allowed us to investigate the influence of electron flow on PSI function. The loss of this electron sink had a profound effect on PSI reaction center properties, including modifications to oligomeric composition, spectral properties of the antenna contained within PSI, and photooxidation capacity. Interestingly, some of these responses were observed even when cells were grown in a standard light regime where ORR1 activity is not thought to play a dominant role.

The most striking consequence to the loss of ORR1 activity was the pronounced increase in the proportion of PSI in the monomeric form. While the overall cellular quantity of PSI was maintained, as well as the ratio of total PSI to PSII, PSI monomers were found to increase 1.4-fold when cells were grown under standard light conditions and 5-fold under increased photon flux, compared with WT. Further investigation of the isolated PSI fractions revealed that all monomeric and trimeric forms from both the WT and ORR1 strains grown under either light regime retained the PsaL subunit. This subunit is necessary for PSI trimer formation and stability,^28,34^ and deletion of this subunit has been extensively utilized for the enrichment and study of PSI monomers.^31,35,36^ While there exists significant diversity in PSI forms across organism taxonomy (monomers, dimers, trimers, tetramers),^37–40^ the purpose for this diversity, what unique properties that each form possesses, and how these might contribute to a role(s) within the larger context of the photosynthetic energy transduction chain are beginning to emerge.^41^ Our work here reveals that diversity in PSI function is also observed as cells adjust to flux, and that a loss or modification of PsaL is not the only means for forming monomers. Therefore, the downstream electron utilization processes are integrated with control over primary photochemical events.

In cyanobacteria, PSI is predominantly found as a trimer. Recently, the location of the red chls in various cyanobacterial trimeric and monomeric PSI complexes has begun to be elucidated,^31,42^ and have shown that one cluster is located at the monomer–monomer interface in close association with PsaL. In *S.* 6803 monomeric complexes devoid of PsaL, there are subsequent spectral changes indicating that the altered environment around the red chls results in a blue shift of the PSI fluorescence emission peak at 77 K (710–715 nm).^31^ This has been shown to be due to the loss of ∼1.5–2 red chls nearby the monomerization region.^31^ Indeed, we observe these changes in our monomeric PSI samples indicating that the environment around PsaL is significantly altered even though this subunit is retained. Interestingly, we also observe alterations in red chl emissive properties in trimeric PSI from ORR1 which become more apparent when cells are grown under increased photon flux. Additionally, the red chls impacted in these samples appear to emerge from the lowest energy states emitting at 722–724 nm. While the precise location of this cluster of red chls has not yet been fully determined, it is likely that they are not located near the monomerization region. These results suggest that PSI trimers undergo perturbations of the protein environment around this cluster of red chls when electron transfer to the ORR pathway is disrupted. The reasons for why PSI from ORR1 strains exhibits such considerable changes in red chl emission will require further biophysical investigations. However, red chls can greatly impact the dynamics of energy transfer and trapping in the PSI core. Energy transfer to P_700_ from red chls is energetically uphill and it has been shown to be slower in monomers *vs.* trimers.^31^ Therefore, the PSI modifications in ORR1 support a slower transfer of excitation energy from the antenna to P_700_. This interpretation is consistent with the diminished ability to keep PSI centers oxidized in ORR1 due to limitations in generating the charge separated state, and partially provides a physical explanation for the observed differences in P_700_ steady state kinetic behavior of both whole cells and isolated PSI complexes in ORR1 compared to WT.

Deviations in red chl emission properties were found to roughly correlate with the light saturation profiles of ORR1 trimers in particular. In the standard light regime, subtle changes to red chl properties resulted in a 10% diminution of the light saturation profile. As the effect on red chl increased when cells were exposed to additional excitation pressure, the light saturation properties were also significantly diminished. There may be additional implications for the interaction with and excitation energy transfer from the light harvesting phycobilisome (PBS) complexes especially since there is evidence for altered molecular interactions of monomeric PSI with PBS.^43–45^ To unravel the contributions from red chl spectral properties on the interaction of PSI with photons will require further investigations.

Although there were changes in the spectral properties, the PSI isolated from either WT or ORR1 strains cultured under normal photon flux had similar rates of Fld reduction, despite the differences in electron utilization between strains. The compensation for loss of ORR1 under these conditions does not appear to include a modification of linear electron flow (LEF) kinetics at PSI but does involve an increase in CEF. This scenario changes in ORR1 under increased photon flux leading to a ∼30% decline in Fld reduction rates mediated by either trimers or monomers compared to WT, as well as a further increased level of CEF. Our work suggests that changes in the photochemical properties of PSI are necessary to compensate for the cumulative effects of the loss of ORR1 and redistribution of electron flow. This work demonstrates how photosynthetic organisms balance photon and electron fluxes by coupling changes in peripheral redox reactions to core PET function, and reveals additional insights into the plasticity of the functional role of PSI.

## 5 Conclusions

There is widespread interest in engineering cyanobacterial systems for the production of solar fuels and chemical compounds. These efforts have included the addition or exchange of carbon utilization pathways, hydrogen producing enzymes and other strategies.^46–51^ While some system engineering approaches were able to adequately substitute one enzyme for another, often there are extraneous effects that limit the outcome and widespread applicability of such methods. Therefore, a molecular level understanding of the intricacies of electron flow and redistribution in response to pathway perturbations is essential to realizing maximal efficiencies in cyanobacterial-based solar fuel production systems. In particular, the impact on reaction center function appears to be a relevant, yet underexplored, variable that could be exploited to improve the rational design of these bioengineering approaches. The success in manipulating model systems requires a more complete understanding of the functional relationships between reaction centers, electron transport and energy utilizing pathways.

## Conflicts of interest

There are no conflicts to declare.

## Abbreviations

*A*_700_
: Absorbance at 700 nm
CEF: Cyclic electron flow
CV: Column volume
DCMU: (3-(3,4-Dichlorophenyl)-1,1-dimethylurea)
FL: Fluctuating light
Fld: Flavodoxin
Flv: Flavodiiron
GL: Growth light
LEF: Linear electron flow
MV: Methyl viologen
ORR: Oxygen reduction reaction
PBS: Phycobilisomes
PET: Photosynthetic electron transport
PSI: Photosystem I
PSII: Photosystem II
Red chls: Long wavelength chlorophylls
TM: Thylakoid membrane
WT: Wild type

## Supplementary Material

RA-012-D2RA01295B-s001
