# Correction: The influence of electron utilization pathways on photosystem I photochemistry in *Synechocystis* sp. PCC 6803

**DOI:** 10.1039/d2ra90130g

**Published:** 2023-01-03

**Authors:** Sharon L. Smolinski, Carolyn E. Lubner, Zhanjun Guo, Jacob H. Artz, Katherine A. Brown, David W. Mulder, Paul W. King

**Affiliations:** National Renewable Energy Laboratory 15013 Denver West Parkway Golden CO 80401 USA paul.king@nrel.gov

## Abstract

Correction for ‘The influence of electron utilization pathways on photosystem I photochemistry in *Synechocystis* sp. PCC 6803’ by Sharon L. Smolinski *et al.*, *RSC Adv.*, 2022, **12**, 14655–14664, https://doi.org/10.1039/d2ra01295b.

The authors regret that in the Experimental section on Page 14657, there are six instances where the abbreviations of micromolar (μM), microliter (μl) and microgram (μg) are incorrectly noted as “mM”, “ml” and “mg”. The corrected units are given below:

Section 2.6. Fluorescence emission analysis

Fractions containing PSI trimers and monomers that were isolated using anion-exchange chromatography were normalized to 3.0 μg chl per ml and were measured at 77 K to quantify P_700_. Fractions containing PSI monomers and trimers that were isolated using sucrose gradients were normalized to 16 μg chl per ml and were measured at room temperature and 77 K to determine spectral properties.

Section 2.7. P_700_ spectroscopic analysis

P_700_ spectroscopic analysis on isolated fractions containing PSI monomers or trimers were normalized to equivalent amounts of P_700_ (0.84 μmol) and measured using 720 nm actinic light. Samples (2 ml) were placed in a quartz cuvette containing P_700_, 10 mM sodium ascorbate, and 10 μM 2,6-dichlorophenolindophenol (DCPIP) in 20 mM HEPES–NaOH, pH 7.5, with 10 mM CaCl_2_, 10 mM MgCl_2_, 10 mM NaCl, and 0.04% DDM.

2.8. Flavodoxin photoreduction assays

In order to determine the capacity of PSI monomers and trimers to transfer electrons out of PSI, equivalent amounts of P_700_ (44 nmol) were added to a quartz cuvette containing 10 mM sodium ascorbate, 30 μM phenazine methosulfate, and 100 μM flavodoxin (Fld), in 20 mM HEPES–NaOH, pH 7.5, with 10 mM CaCl_2_, 10 mM MgCl_2_, 10 mM NaCl, and 0.04% DDM, at a final volume of 350 μl, similar to ref. 26.

The Royal Society of Chemistry apologises for these errors and any consequent inconvenience to authors and readers.

## Supplementary Material

