# Supplementary material for: The influence of electron utilization pathways on photosystem I photochemistry in Synechocystis sp. PCC 6803
Source: RSC Adv. 2022 May 16;12(23):14655–64. doi: 10.1039/d2ra01295b (PMC9109680; doi:10.1039/d2ra01295b)
Supplement: RA-012-D2RA01295B-s001 [file RA-012-D2RA01295B-s001.pdf]

Electronic Supplementary Material (ESI) for RSC Advances.
This journal is © The Royal Society of Chemistry 2022

Supplementary Information for

The Influence of Electron Utilization Pathways on Photosystem I Photochemistry in *Synechocystis* sp. PCC 6803

Sharon L. Smolinski, Carolyn E. Lubner, Zhanjun Guo, Jacob H. Artz, Katherine A. Brown, David W. Mulder, and Paul W. King

Corresponding Author: Paul W. King, paul.king@nrel.gov

This PDF file includes:

Supplementary Experimental
Figures S1 to S10
Tables S1 to S10

Table of Contents

Supplementary Experimental ... 1-2
Figure S1 ... 3
Figure S2 ... 4
Figure S3 ... 5
Figure S4 ... 6
Figure S5 ... 7
Figure S6 ... 8
Figure S7 ... 9
Figure S8 ... 10
Figure S9 ... 11
Figure S10 ... 12
Table 1 ... 13
Table 2 ... 14
Table 3 ... 15
Table 4 ... 16
Table 5 ... 17
Table 6 ... 18
Table 7 ... 19
Table 8 ... 20
Table 9 ... 21
Table 10 ... 22

Supplementary Experimental

O_2 uptake

O_2 uptake activities from *S.* 6803 cell suspensions grown in either GL or FL were measured at the final growth timepoint, with an HPR-40 MIMS Membrane Inlet Mass Spectrometer (Hiden Analytical, United Kingdom) equipped with a quartz probe. The probe was immersed in a 0.01 L custom-made transparent gas-tight glass reaction vessel (Allen Scientific Glass Blowers, USA). The reaction vessel was placed in a black PVC box opaque to light, which was equipped with 4 2V-LED (one on each side of the box) providing a cool white light (6000 K). Highest intensity inside the reaction vessel was estimated to be 400 μmol photons m^{-2} s^{-1}.

4 ml of fresh BG-11 medium was first introduced into the reaction vessel, continuously stirred and kept at 30 °C by a thermostatic water bath surrounding the reaction vessel. N_2 was bubbled for 3 min in the closed system to remove most of the $^{16}O_2$. A controlled volume of $^{18}O_2$ was then injected in the system; equilibration between the headspace and the liquid phase was achieved after 10-15 min. A 2 mL aliquot of cell sample was placed in a sealed vial and flushed with N_2 for 2 min in the dark and then injected into the reaction vessel. After 3 min of equilibration in the dark, samples were illuminated with white light (400 μmol photons m^{-2} s^{-1}). The reaction vessel was washed between experiments with ethanol and thoroughly rinsed with deionized water. $^{16}O_2$ evolution and $^{18}O_2$ uptake rates were determined by fitting the initial change in concentration to a linear function. Rates were then normalized to chlorophyll (chl) concentration. Chl concentration was determined by preparing 10 μl of sample in 990 μl methanol, vortexing and then centrifuging the sample at 6,200 x *g* for 1 min. Absorbance of the supernatant was measured at 663 nm, and chl concentration was determined from the equation: mg ml^{-1} = A663 x (100/82).

SDS-PAGE and immunoblotting

In order to assess the PsaL protein levels across the purified fractions from DEAE column, samples, normalized to the same chl concentration, were incubated with equal volume of 2X Laemmli SDS sample buffer containing 5% 2-mercaptoethanol at 100 °C for 10 min. Samples were loaded onto Tris-Glycine gels (4568124, BioRad), alongside standards (1610376, BioRad) and run at 200 V for 30 min. The proteins were transferred to a polyvinylidene fluoride (PVDF) membrane (1620174, BioRad), using a semidry apparatus, and immunoblotted with PsaL antibody (provided by Dr. John Golbeck's laboratory, Penn State). The original image was reversed in order to show black bands against a white background and cropped.

Determination of P_{700}

The amount of P_{700} in the PSI trimers and monomers isolated using anion-exchange chromatography was determined using fluorescence emission analysis at 77K (**Table S**). The chl concentration of the samples were normalized to 3 mg ml^{-1}. The maximum value of the peaks at approximately 718 nm and 685 nm, associated with PSI and PSII respectively, were determined. The ratio of the chl attributable to PSI and PSII was determined by maximum value of peak at 718 nm ÷ maximum value of peak at 685 nm. The P_{700} (nmol) in the PSI samples was determined based on the amount of chl in the sample attributable to PSI. In the diluted PSI trimer samples, P_{700} (nmol) was determined by: (chl concentration of diluted sample ÷ 893.51) ÷ 95. P_{700} in the diluted PSI monomer samples (nmol) was determined by: [(chl concentration of diluted sample x (ratio for PSI:PSII x 95)) ÷ ((ratio for PSI:PSII x 95) + (ratio value for PSII, which is 1 x 35) ÷ 893.51] ÷ 95. The number of chl molecules in PSI monomeric unit: 95. The number of chl molecules in PSII: 35. The molecular weight of chl: 893.51 g mol^{-1}. The amount of P_{700} (nmol) in the non-diluted PSI monomer or trimer sample (nmol) based on the amount of chl, as determined by: [(nmol P_{700} in diluted sample for fluorescence emission spectra samples) ÷ (3 mg ml^{-1} chl)] x (chl concentration of non-diluted PSI sample).

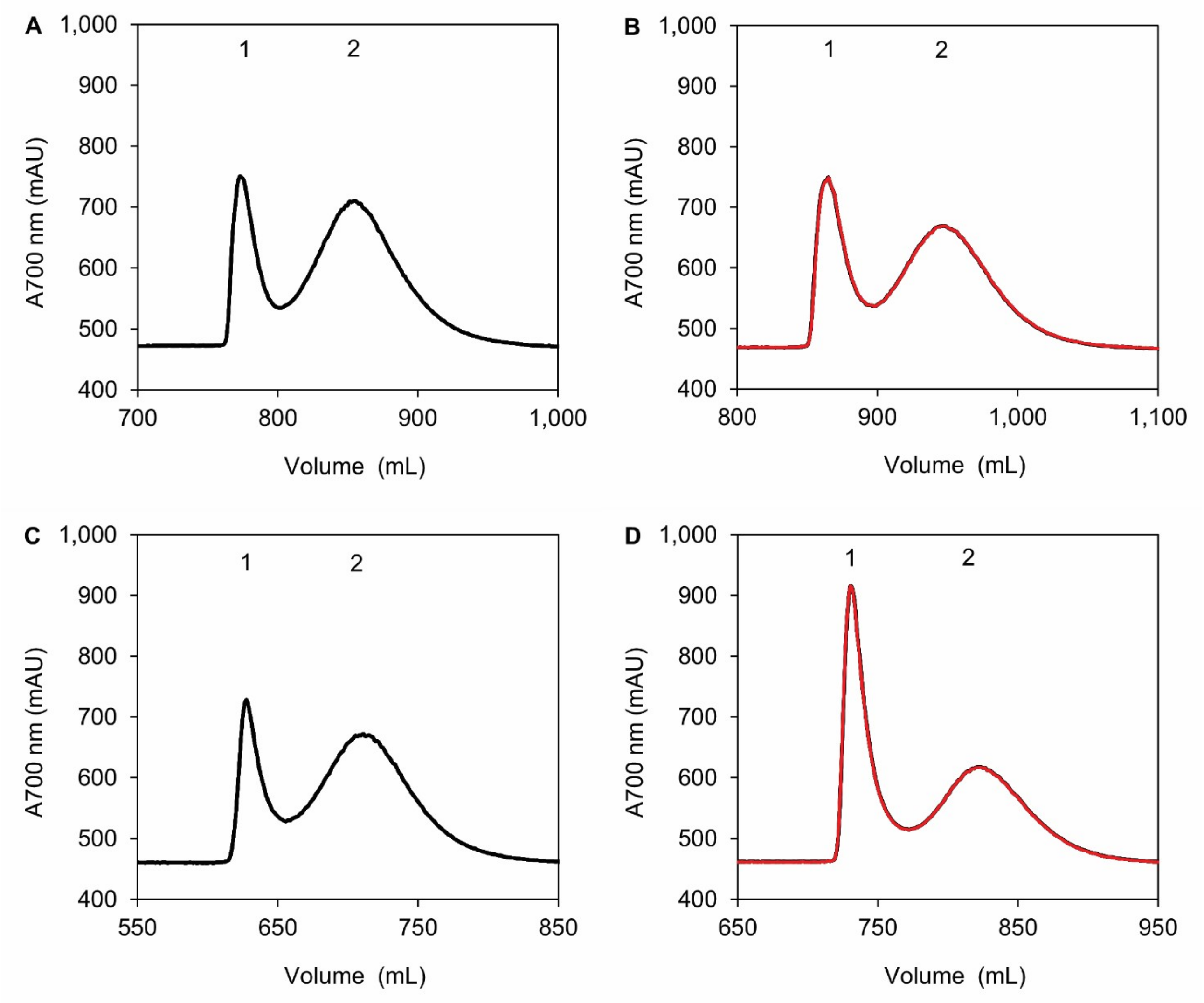

Fig. S1. Elution profiles from anion exchange chromatography showing two fractions isolated from thylakoid membranes. A) WT cultured in standard conditions for 96 h, B) ORR1 cultured in standard conditions for 96 h, C) WT cultured under increased flux (FL) for 96 h, D) ORR1 cultured under increased flux (FL) for 96 h. Peaks showing absorbance at 700 nm correspond to fraction 1 containing PSI monomers (and PSII), and fraction 2 containing PSI trimers. Volume is total eluted volume, with 0 mL being the point at which sample was loaded. WT: black; ORR1: red.

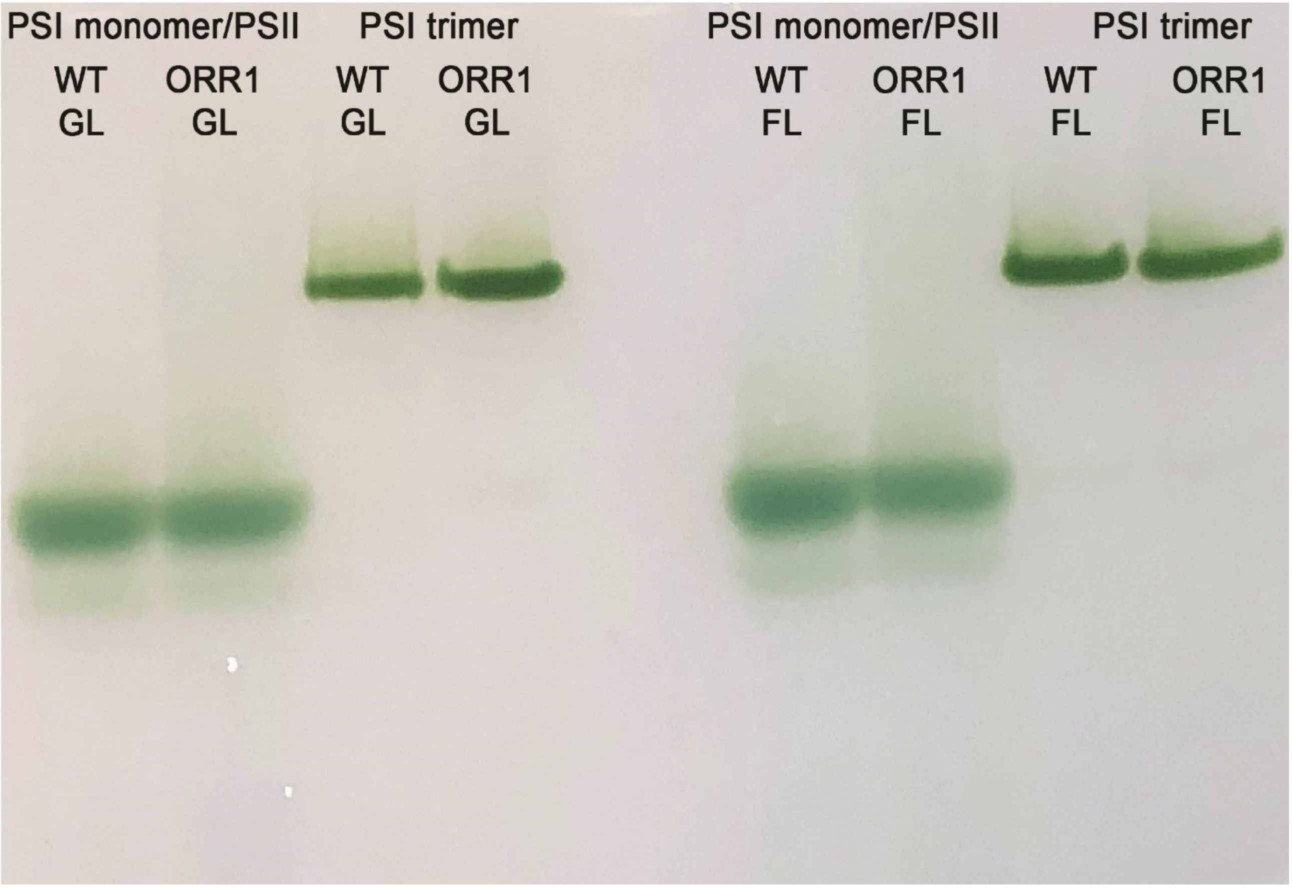

Fig. S2. BN-PAGE analysis of fractions containing PSI monomers or trimers. Fractions were purified from thylakoid membranes using sucrose gradients, from WT and ORR1 cells grown in standard conditions (GL) or increased photon flux (FL) for 96 h.

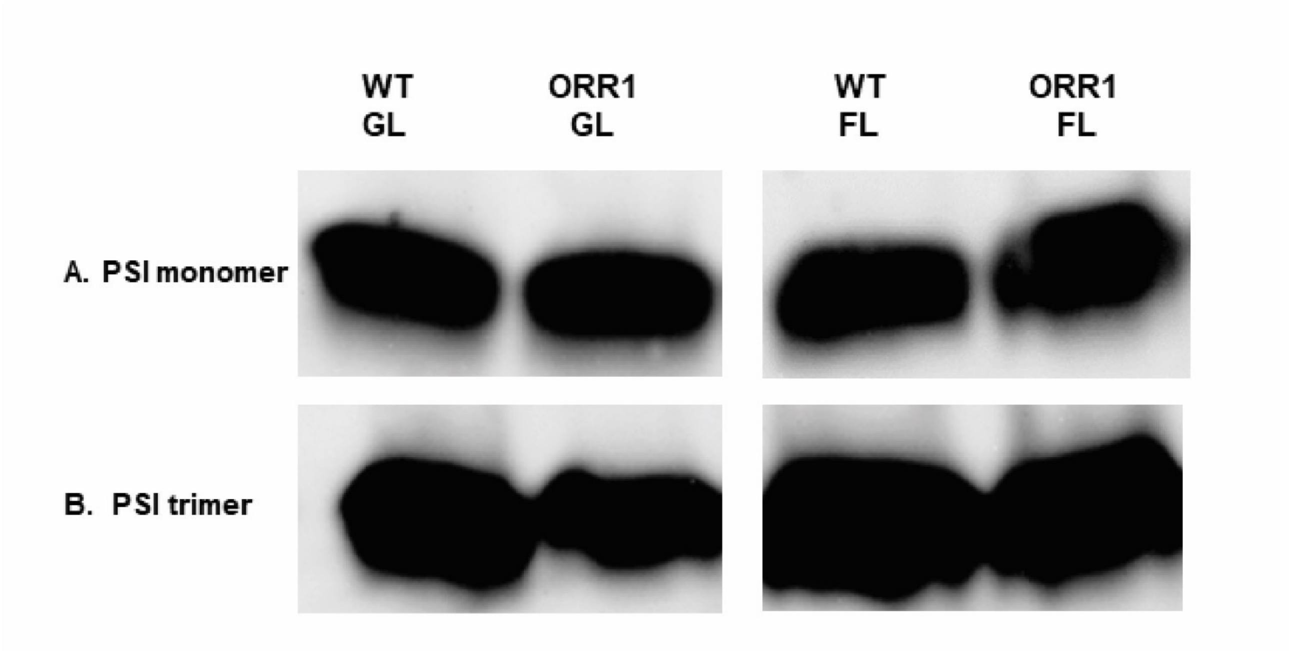

Fig. S3. Immunoblots of isolated PSI monomers and trimers from cells cultured under standard growth conditions (GL) and increased and fluctuating light (FL). PSI monomers or trimers were isolated using anion-exchange chromatography, separated using SDS-PAGE and immunoblotted against PsaL antibody: A) PSI monomers, B) PSI trimers.

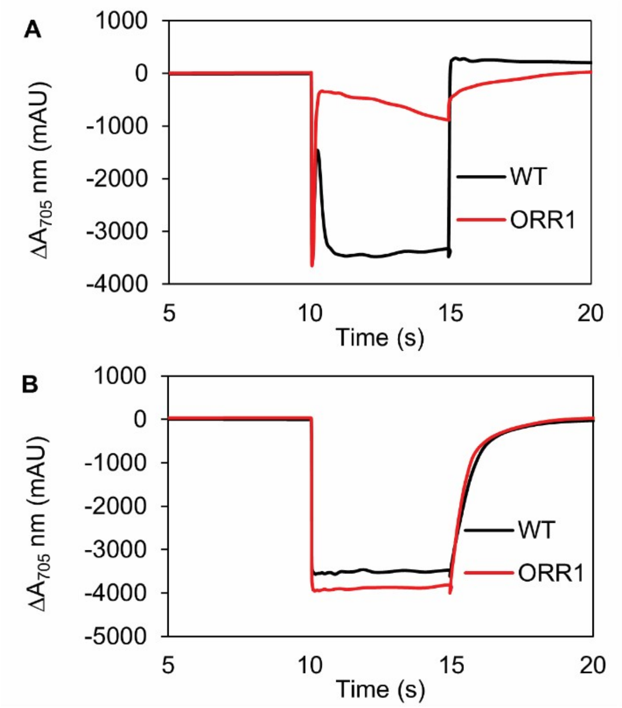

Fig. S4. P_{700} kinetics on whole cells cultured in standard conditions (GL) for 96 h, and measured A) without inhibitors, and B) upon the addition of DCMU. WT: black; ORR1: red. Data are the average of 3 to 4 biological replicates.

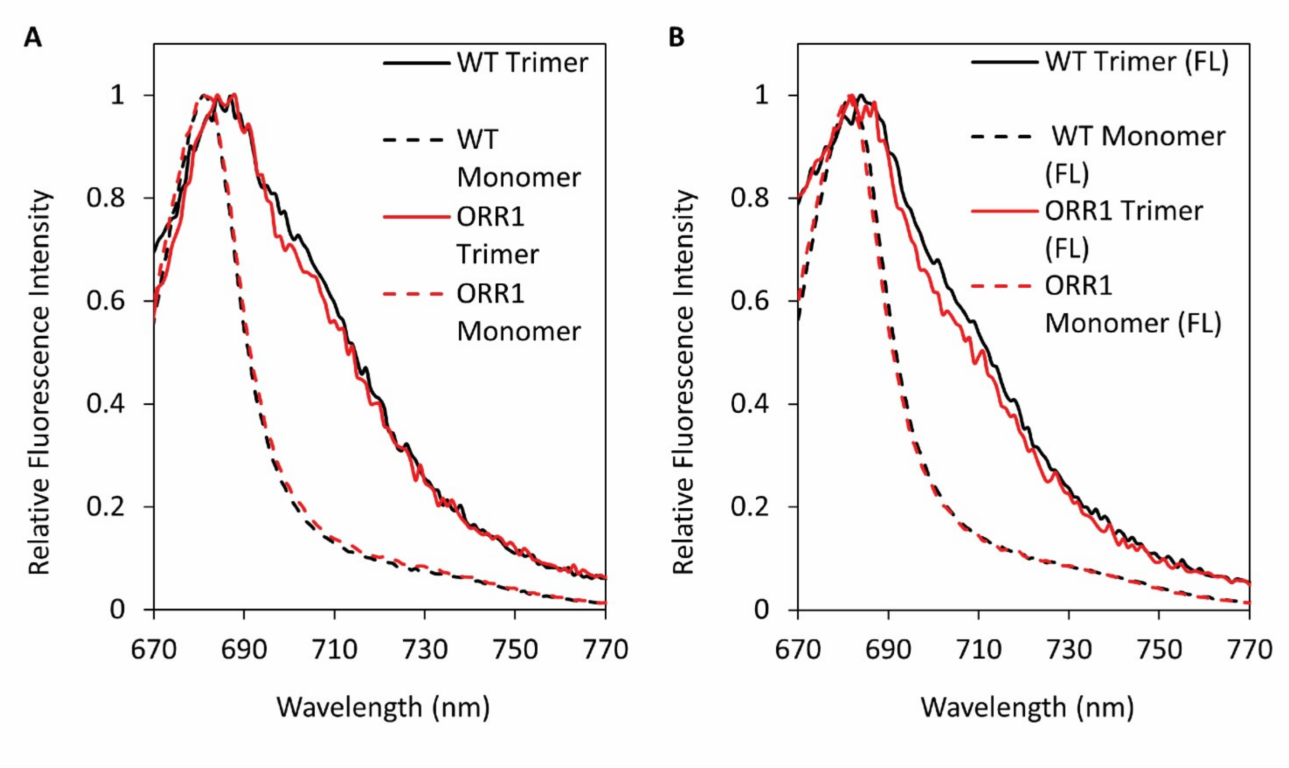

Fig. S5. Fluorescence emission spectra of PSI monomers and trimers. A) Fluorescence emission spectra collected at room temperature using 450 nm excitation on PSI trimers and monomers isolated from WT and ORR1 cells grown in standard conditions. B) Fluorescence emission spectra collected at room temperature using 450 nm excitation on PSI trimers and monomers isolated from cells grown in increased photon flux conditions (FL). Peak intensity was normalized to WT trimer for each condition. WT: black, ORR1: red, PSI trimers: solid line, PSI monomers: dashed line.

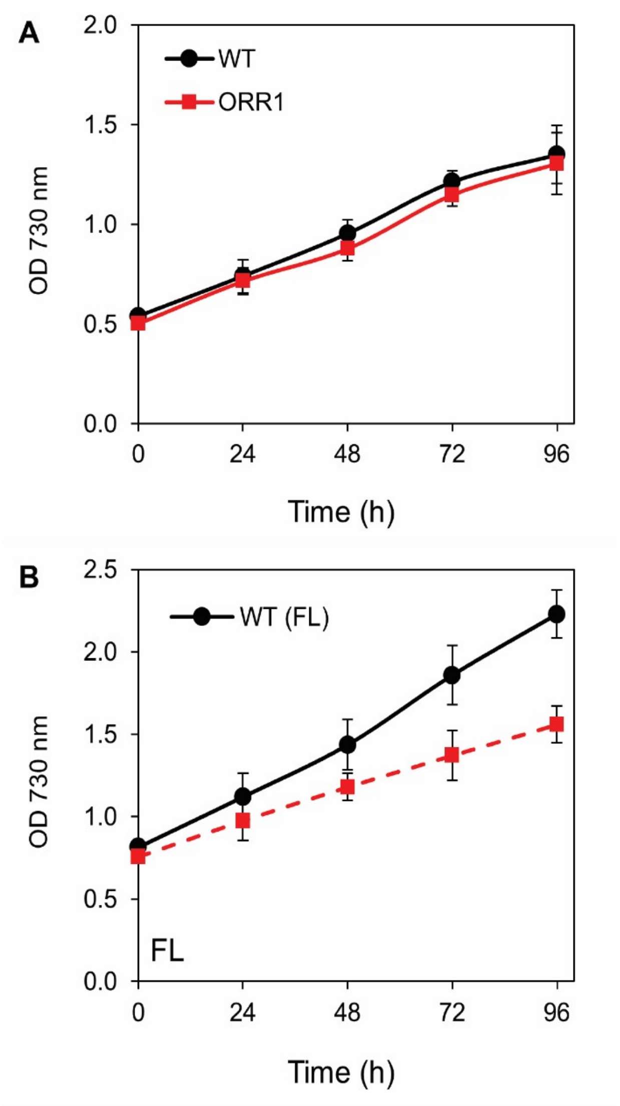

Fig. S6. Growth phenotype of strains in A) standard conditions and B) under increased flux (FL). WT: black; ORR1: red. Data are based on the average of 6-8 biological replicates ± standard deviation.

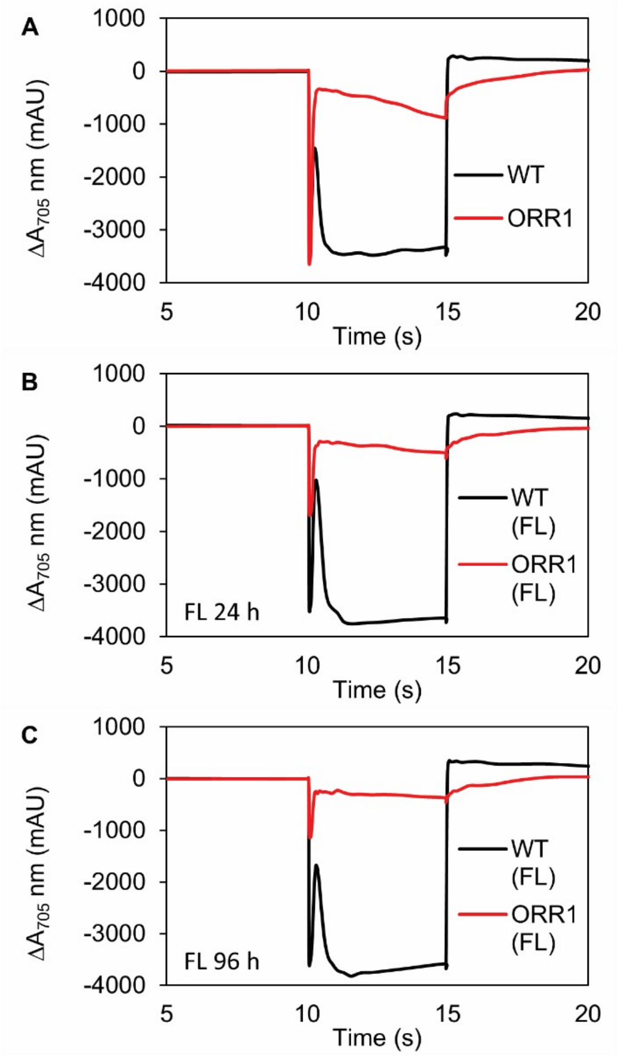

Fig. S7. P_{700} kinetics of whole cells after growth in A) standard conditions for 96 h, and B) 24 h and C) 96 h increased flux (FL). WT: black, ORR1: red. Data are the average of 4 biological replicates.

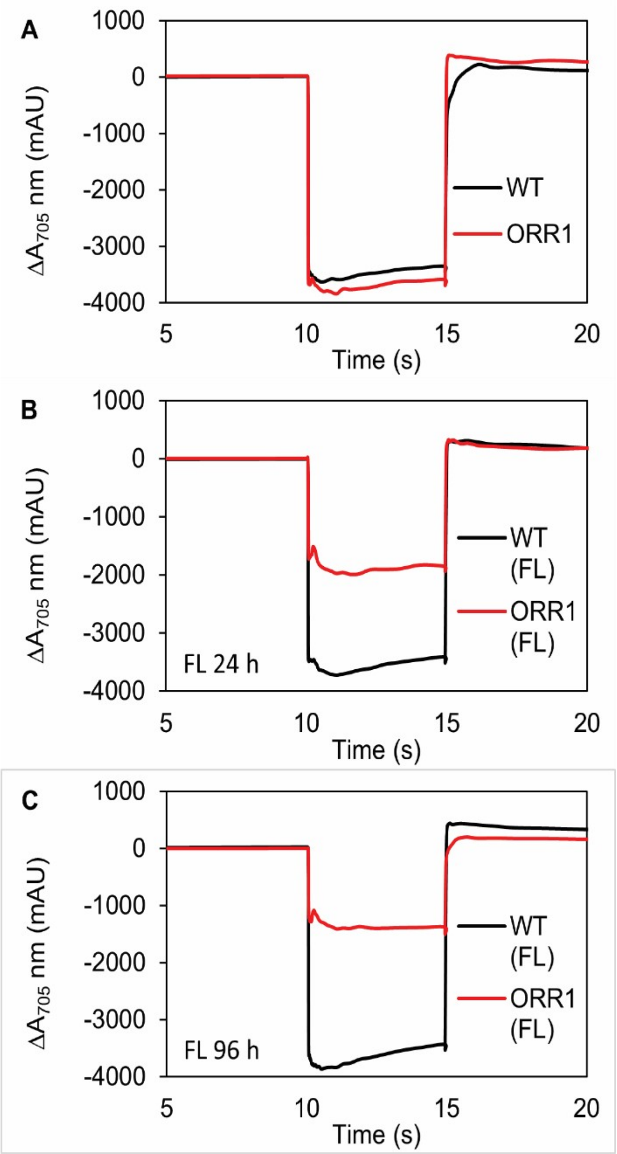

Fig. S8. P_{700} kinetics on whole cells upon addition of MV after growth in A) standard conditions for 96 h, and after B) 24 h and C) 96 h under increased flux (FL). Data are the average of 3 to 4 biological replicates. WT: black; ORR1: red.

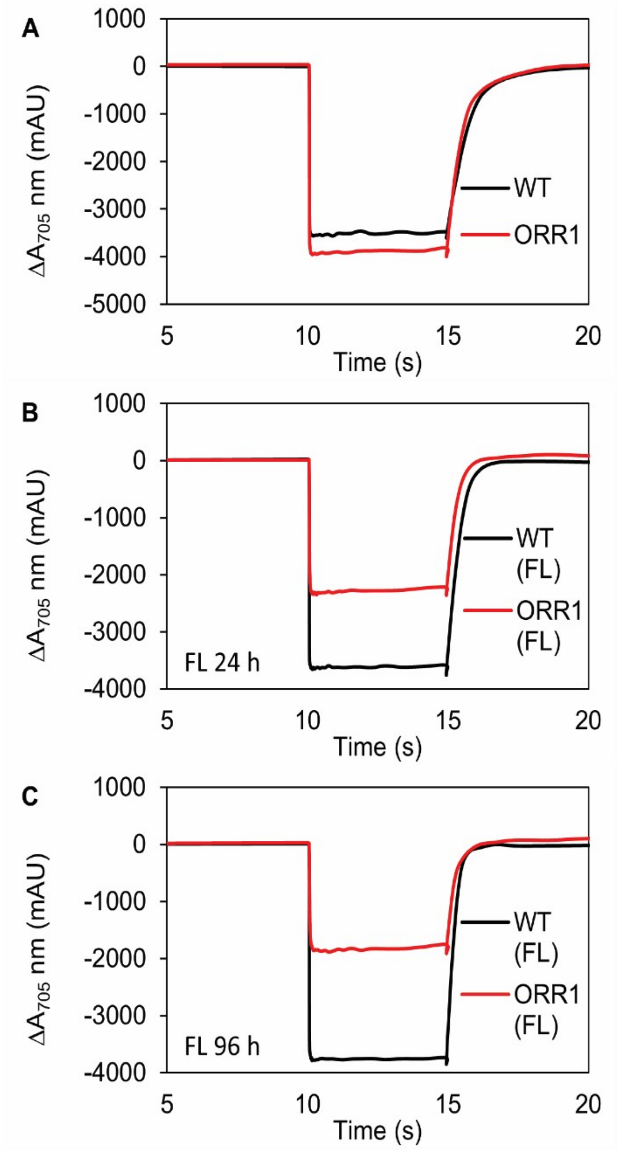

Fig. S9. P_{700} kinetics on whole cells measured upon the addition of DCMU after growth in A) standard conditions for 96 h, and B) 24 h and C) 96 h under increased flux (FL). Data are the average of 3 to 4 biological replicates. WT: black; ORR1: red.

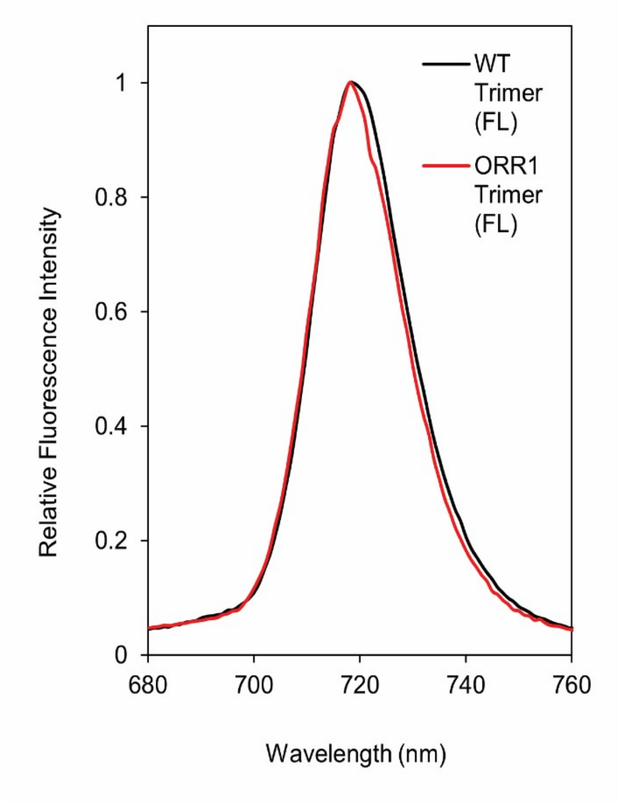

Fig. S10. 1 Fluorescence emission spectra of PSI trimers collected at 77K using 450 nm excitation on FL PSI trimers. Data were normalized to the maximum peak (718 nm). Peak intensity was normalized to WT trimer for each condition. WT: black, ORR1: red, standard growth conditions: transparent line, FL: solid line.

Table S1. Primers used for construction of the ORR1 strain.

Target Fragment	Primer Name	Sequence
flv1 left arm	5’EcoRI-flv1F	GAATTCCTCCTGGCCCTCAAAG
	3’flv1/aadA-atgR	ACCGCTTCCCTCATTGTTAACTAACC
flv1 right arm	5’flv1/aadA-taaF	GGTAGTCGGCAAATAATTCAATGCGC
	3’EcoRI-flv1R	GAATTCCAACGGTGCTAACAACAAG
Sp cassette	5’flv1/aadA-atgF	GGTTAGTTAACAATGAGGGAAGCGGT
	3’flv1/aadA-taaR	GCGCATTGAATTATTTGCCGACTACC

Table S2. Data used to calculate the ratio of PSI trimer to PSI monomer in thylakoid membrane samples.

Growth Condition	Strain	Biological Replicate	Total A_{700} Peak Area PSI Monomer + PSII[a]	Fraction of Chl 77K Emission from PSI Monomers[b]	PSI Monomer A_{700} Peak Area[c]	PSI Trimer A_{700} Peak Area[d]	PSI Trimer: PSI Monomer[e]	PSI Monomer: PSI Trimer[f]
GL	WT	1	5939	0.35	2079	17654	8.5	0.12
		2	7552	0.45	3398	16654	4.9	0.20
		3	6341	0.44	2790	15121	5.4	0.18
	ORR1	1	6634	0.47	3118	16289	5.2	0.19
		2	7088	0.45	3190	17212	5.4	0.19
		3	7996	0.54	4318	14312	3.3	0.30
FL	WT	1	5643	0.35	1975	16671	8.4	0.12
		2	5556	0.35	1945	16826	8.7	0.12
		3	5460	0.33	1802	18857	10.5	0.10
	ORR1	1	10722	0.68	7291	10468	1.4	0.70
		2	9592	0.60	5755	12039	2.1	0.48
		3	10093	0.54	5450	13560	2.5	0.40

Values based on 2 mg chl of thylakoid membrane samples loaded onto DEAE column and separated by anion-exchange chromatography.

[a] Peak areas of absorbance at 700 nm (A_{700}) for the fraction containing PSI monomer and PSII from the separation of TM using anion exchange chromatography, based in the integration of peaks using Unicorn software (v. 5.2).

[b] Calculated as the $\varepsilon_{720} \div (\varepsilon_{720} + \varepsilon_{686})$ from the 77K fluorescence spectra using excitation at 450 nm. ε_{720} nm and ε_{686} nm are assigned to PSI monomers and PSII, respectively.

[c] Fraction of A_{700} from PSI monomers, calculated as (Total A_{700} Peak Area) × (Fraction of Chl 77K emission from PSI monomers).

[d] Peak area of A_{700} for PSI trimer based on the isolation of PSI trimer using anion exchange chromatography, based on the integration of peaks using Unicorn software (v. 5.2.

[e] Ratio of PSI trimer to PSI monomer, calculated as (PSI Trimer A_{700} Peak Area) ÷ (PSI Monomer A_{700} Peak Area).

[f] Ratio of PSI monomer to PSI trimer, calculated as (PSI Monomers A_{700} Peak Area) ÷ (PSI Trimer A_{700} Peak Area).

Table S3. Data used to calculate the percentage of PSI trimer and PSI monomer in thylakoid membrane samples.

Growth Condition	Strain	Biological Replicate	PSI Monomer A_{700} Peak Area[a]	PSI Trimer A_{700} Peak Area[b]	PSI Trimer (% of total PSI)[c]	PSI Monomer (% of total PSI)[d]
GL	WT	1	2062	17654	89	11
		2	3399	16654	83	17
		3	2790.0	15121	84	16
	ORR1	1	3118	16289	84	16
		2	3190	17212	84	16
		3	4318	14312	77	23
FL	WT	1	1975	16671	89	11
		2	1945	16826	90	10
		3	1802	18857	91	9
	ORR1	1	7291	10468	59	41
		2	5755	12039	68	32
		3	5349	13560	71	29

Values based on 2 mg chl of thylakoid membrane samples loaded onto DEAE column and separated by anion-exchange chromatography.

[a] Fraction of A_{700} from PSI monomers, calculated as (Total A_{700} Peak Area) × (Fraction of Chl 77K emission from PSI monomers), see Table S1.

[b] Peak area of A_{700} for PSI trimer based on the isolation of PSI trimer using anion exchange chromatography, based on the integration of peaks using Unicorn software (v. 5.2), see Table S1.

[c] PSI trimer as a percentage of the total PSI based on areas of fractions, calculated as (Peak area of PSI trimer) ÷ (Peak area of PSI trimer + Peak area of PSI monomer) X 100.

[d] PSI monomer as a percentage of the total PSI based on areas of fractions, calculated as (Peak area of PSI monomer) ÷ (Peak area of PSI monomer + Peak area of PSI trimer) X 100.

Table S4. $^{18}O_2$ uptake and $^{16}O_2$ evolution in WT and ORR1 cells grown under standard conditions.

Strain	$^{18}O_2$ Uptake[a]	$^{16}O_2$ Evolution[b]
WT	0.8 ± 0.4	11.2 ± 0.4
ORR1	0	6.3 ± 0.2

[a] Rates of $^{16}O_2$ evolution as ppm s^{-1} μg Chl^{-1} were determined by fitting the initial change in concentration to a linear function, and then normalized to chlorophyll (chl) concentration. Values are based on two measurements.

[b] Rates of $^{18}O_2$ uptake as ppm s^{-1} μg Chl^{-1} were determined by fitting the initial change in concentration to a linear function, and then normalized to chlorophyll (chl) concentration. Values are based on two measurements.

Table S5. Ratio of PSI to PSII in WT and ORR deficient strains based on 77 K fluorescence.

Light Regime	Strain	PSI:PSII (77K)
GL	WT	4.7 ± 0.2
	ORR1	4.9 ± 0.5
FL	WT	4.5 ± 0.2
	ORR1	4.3 ± 0.2

Table S6. P_{700} photooxidation of PSI monomers and trimers from WT and ORR1 measured at different intensities of actinic light. Cells were grown under standard (GL) or increased photon flux (FL) conditions.

Growth Condition	Actinic Light (μmol photons m^{-2} s^{-1})	A705 nm (mAU as % of WT maximum value) [a]			
		WT PSI Trimer	ORR1 PSI Trimer	WT PSI Monomer	ORR1 PSI Monomer
GL	250	0.021[b] (39)	0.020 (35)	0.023[b] (39)	0.027 (47)
	500	0.035 (63)	0.028 (52)	0.037 (64)	0.038 (66)
	530	0.035 (64)	0.029 (53)	0.038 (66)	0.039 (67)
	550	0.036 (65)	0.030 (54)	0.039 (67)	0.039 (68)
	1000	0.044 (80)	0.037 (67)	0.047 (82)	0.047 (80)
	2000	0.050 (92)	0.042 (76)	0.054 (93)	0.052 (89)
	2500	0.052 (94)	0.043 (79)	0.055 (95)	0.052 (90)
	3000	0.053 (96)	0.044 (80)	0.056 (97)	0.053 (92)
	4000	0.054 (99)	0.045 (83)	0.057 (99)	0.054 (93)
	4500	0.054 (99)	0.046 (83)	0.058 (100)	0.054 (94)
	5000	0.055 (100)	0.046 (84)	0.058 (100)	0.055 (94)
FL	250	0.019 (40)	0.011 (22)	0.020 (39)	0.009 (19)
	500	0.028 (59)	0.015 (30)	0.029 (59)	0.014 (29)
	530	0.029 (60)	0.015 (31)	0.030 (61)	0.014 (29)
	550	0.030 (61)	0.018 (31)	0.031 (62)	0.015 (30)
	1000	0.037 (77)	0.020 (38)	0.039 (78)	0.018 (36)
	2000	0.044 (90)	0.021 (42)	0.045 (91)	0.020 (41)
	2500	0.045 (93)	0.021 (43)	0.046 (93)	0.021 (42)
	3000	0.046 (95)	0.021 (43)	0.047 (96)	0.021 (42)
	4000	0.048 (98)	0.022 (45)	0.049 (99)	0.022 (44)
	4500	0.048 (99)	0.022 (45)	0.049 (99)	0.022 (44)
	5000	0.049 (100)	0.022 (45)	0.050 (100)	0.022 (44)

[a] Maximum P_{700} oxidation values are absorbance at 705 nm, at 15.59 ms near the end of illumination.

[b] WT PSI trimer and monomer (GL) measured at 200 μmol photons m^{-2} s^{-1}.

Table S7. Maximum P_{700} oxidation measured in WT and ORR1 whole cells without the addition of inhibitors and upon the addition of MV or DCMU.

Inhibitor	Growth Condition	Strain	A705 nm mAU (% of WT) [a]		
			0 h[b]	24 h[c]	96 h[d]
None	GL	WT	4040 ± 530 (100)	3381 ± 663 (100)	3394.8 ± 158 (100)
		ORR1	750 ± 29 (19)	678 ± 106 (20)	786.0 ± 285 (23)
	FL	WT	3891 ± 348 (100)	3669 ± 73 (100)	3641.7 ± 139 (100)
		ORR1	690 ± 41 (18)	443 ± 68 (12)	324.1 ± 38 (9)
DCMU	GL	WT	4170 ± 662 (100)	3464 ± 731 (100)	3536 ± 251 (100)
		ORR1	3708 ± 345 (89)	3869 ± 180 (112)	3912 ± 198 (111)
	FL	WT	3787 ± 541 (100)	3620 ± 78 (100)	3756 ± 243 (100)
		ORR1	3518 ± 316 (93)	2240 ± 254 (62)	1801 ± 145 (48)
MV	GL	WT	3711 ± 599 (100)	3201 ± 715 (100)	3348 ± 142 (100)
		ORR1	3391 ± 382 (91)	3574 ± 174 (112)	3621 ± 114 (108)
	FL	WT	3521 ± 389 (100)	3461 ± 80 (100)	3496 ± 227 (100)
		ORR1	3365 ± 313 (96)	1833 ± 303 (53)	1392 ± 233 (40)

[a] Maximum P_{700} oxidation values are absorbance at 705 nm, at 14.07 ms near the end of illumination, measured using 2000 μmol photons m^{-2} s^{-1} actinic light and equivalent chl. Values based on three to four biological replicates per strain and condition.

[b] Whole cells measured after an initial 36 h of grown in standard conditions (GL).

[c] Whole cells measured after 24 h in standard conditions (GL) or under increased photon flux (FL), following 36 h of initial growth in standard conditions.

[d] Whole cells measured after 96 h in standard conditions (GL) or under increased photon flux (FL), following 36 h of initial growth in standard conditions.

S8. Rates of P_{700} dark re-reduction measured in WT and ORR1 whole cells without the addition of inhibitors and upon the addition of DCMU. Data were collected from whole cells grown in standard conditions (GL) or increased photon flux (FL) for 96 h, and based on measurements using 2000 μmol photons m^{-2} s^{-1} actinic light and equivalent chl.

Inhibitor	Growth Condition	Strain	Half-life of dark re-reduction (ms)[a]
None	GL	WT	0.01 ± 0.005
		ORR1	563 ± 183
	FL	WT	0.01 ± 0.001
		ORR1	570 ± 258
DCMU	GL	WT	384 ± 87
		ORR1	215 ± 29
	FL	WT	183 ± 50
		ORR1	130 ± 6

[a] Values were calculated as half-life in milliseconds. The half-life of dark re-reduction (ms) was determined using either a linear (no inhibitor WT data) or a single exponential equation (for no inhibitor ORR strain data and DCMU data). Data are the average of two (ORR1 GL), or three to four biological replicates ± standard deviation.

Table S9. $^{18}O_2$ uptake, $^{16}O_2$ evolution in cells in WT and ORR1 cells harvested after 96 h of growth under FL.

Strain	$^{18}O_2$ Uptake[a]	$^{16}O_2$ Evolution[b]
WT	1.9 ± 0.2	10.6 ± 0.2
ORR1	0	8.7 ± 0.1

[a] Rates of $^{16}O_2$ evolution as ppm s^{-1} μg Chl^{-1} were determined by fitting the initial change in concentration to a linear function, and then normalized to chlorophyll (chl) concentration. Values are based on single measurements.

[b] Rates of $^{18}O_2$ uptake as ppm s^{-1} μg Chl^{-1} were determined by fitting the initial change in concentration to a linear function, and then normalized to chlorophyll (chl) concentration. Values are based on single measurements.

Table S10. Quantification of P_{700} in samples containing PSI trimer and monomer.

Growth Condition	Strain	PSI[a]	Chl[b]	Max value PSI peak[c]	Max value PSII peak[d]	PSI:PSII[e]	P_{700} in sample for emission analysis[f]	P_{700} in stock sample[g]
GL	WT	Trimer	235	42597			35	2769
		Trimer	76	64023			35	894
	ORR1	Trimer	206	47630			35	2427
		Trimer	293	69683			35	3452
	WT	Monomer	126	32657	74507	0.44	19	806
		Monomer	51	69333	178057	0.39	18	306
	ORR1	Monomer	238	45590	82700	0.55	21	1681
		Monomer	83	83757	128610	0.65	23	622
FL	WT	Trimer	225	59000			35	2641
		Trimer	98	33123			35	1155
	ORR1	Trimer	232	71893			35	2733
		Trimer	256	56660			35	3016
	WT	Monomer	125	56107	118763	0.47	20	827
		Monomer	101	59093	152363	0.39	18	610
	ORR1	Monomer	169	65703	84453	0.78	24	1351
		Monomer	75	104900	152073	0.69	23	577

[a] Samples containing PSI trimer and monomer were isolated from thylakoid membranes using anion exchange chromatography, from cells cultured for 96 h under standard conditions (GL) or under increased photon flux (FL).

[b] Chl concentration based on A_{663} nm by diluting 10 µl sample in 990 µl methanol, A_{663} x (100 ÷ 82).

[c] Maximum value of peak at approximately 718 nm, associated with PSI, as determined by fluorescence emission spectra at 77K using 450 nm excitation wavelength and equivalent chl (3 µg ml^{-1}).

[d] Maximum value of peak at approximately 685 nm, associated with PSII, as determined by fluorescence emission spectra at 77K using 450 nm excitation wavelength and equivalent chl (3 µg ml^{-1}).

[e] Ratio of the maximum values of peak at 718 nm to 685 nm, which is the ratio of chl in PSI to chl in PSII, based on fluorescence emission spectra at 77K.

[f] P_{700} (nmol) in the PSI samples was based on the amount of chl in the sample attributable to PSI. In the diluted PSI trimer samples, P_{700} (nmol) was determined by: (chl concentration of diluted sample ÷ 893.51) ÷ 95. P_{700} in the diluted PSI monomer samples (nmol) was determined by: [(chl concentration of diluted sample x (ratio for PSI:PSII x 95)) ÷ ((ratio for PSI:PSII x 95) + (ratio value for PSII, which is 1 x 35) ÷ 893.51] ÷ 95. The number of chl molecules in PSI monomeric unit: 95. The number of chl molecules in PSII: 35. The molecular weight of chl: 893.51 g mol^{-1}.

[g] P_{700} in the non-diluted PSI monomer or trimer sample (nmol) based on the amount of chl, as determined by: [(nmol P_{700}^{+} in diluted sample for fluorescence emission spectra samples) ÷ (3 µg ml-1 chl)] x (chl concentration of non-diluted PSI sample).
